# *APOE*-related risk of mild cognitive impairment and dementia for prevention trials: An analysis of four cohorts

**DOI:** 10.1371/journal.pmed.1002254

**Published:** 2017-03-21

**Authors:** Jing Qian, Frank J. Wolters, Alexa Beiser, Mary Haan, M. Arfan Ikram, Jason Karlawish, Jessica B. Langbaum, John M. Neuhaus, Eric M. Reiman, J. Scott Roberts, Sudha Seshadri, Pierre N. Tariot, Beth McCarty Woods, Rebecca A. Betensky, Deborah Blacker

**Affiliations:** 1 Department of Biostatistics and Epidemiology, University of Massachusetts Amherst, Amherst, Massachusetts, United States of America; 2 Department of Epidemiology, Erasmus Medical Center, Rotterdam, the Netherlands; 3 Department of Neurology, Boston University Medical School, Boston, Massachusetts, United States of America; 4 Department of Biostatistics, Boston University School of Public Health, Boston, Massachusetts, United States of America; 5 Department of Epidemiology and Biostatistics, University of California, San Francisco, San Francisco, California, United States of America; 6 Department of Medicine, University of Pennsylvania Medical School, Philadelphia, Pennsylvania, United States of America; 7 Banner Alzheimer’s Institute, Phoenix, Arizona, United States of America; 8 Department of Psychiatry, University of Arizona College of Medicine, Phoenix, Arizona, United States of America; 9 Arizona State University–Banner Neurodegenerative Disease Research Center, Tempe, Arizona, United States of America; 10 Neurogenomics Division, Translational Genomics Research Institute, Phoenix, Arizona, United States of America; 11 Department of Health Behavior & Health Education, University of Michigan School of Public Health, Ann Arbor, Michigan, United States of America; 12 Department of Biostatistics, Harvard T.H. Chan School of Public Health, Boston, Massachusetts, United States of America; 13 Department of Psychiatry, Massachusetts General Hospital/Harvard Medical School, Boston, Massachusetts, United States of America; 14 Department of Epidemiology, Harvard T.H. Chan School of Public Health, Boston, Massachusetts, United States of America; University of California San Francisco Memory and Aging Center, UNITED STATES

## Abstract

**Background:**

With the onset of prevention trials for individuals at high risk for Alzheimer disease, there is increasing need for accurate risk prediction to inform study design and enrollment, but available risk estimates are limited. We developed risk estimates for the incidence of mild cognitive impairment (MCI) or dementia among cognitively unimpaired individuals by *APOE*-e4 dose for the genetic disclosure process of the Alzheimer’s Prevention Initiative Generation Study, a prevention trial in cognitively unimpaired *APOE*-e4/e4 homozygote individuals.

**Methods and findings:**

We included cognitively unimpaired individuals aged 60–75 y, consistent with Generation Study eligibility criteria, from the National Alzheimer’s Coordinating Center (NACC) (*n* = 5,073, 158 *APOE-*e4/e4), the Rotterdam Study (*n* = 6,399, 156 *APOE-*e4/e4), the Framingham Heart Study (*n* = 4,078, 67 *APOE-*e4/e4), and the Sacramento Area Latino Study on Aging (SALSA) (*n* = 1,294, 11 *APOE-*e4/e4). We computed stratified cumulative incidence curves by age (60–64, 65–69, 70–75 y) and *APOE-*e4 dose, adjusting for the competing risk of mortality, and determined risk of MCI and/or dementia by genotype and baseline age. We also used subdistribution hazard regression to model relative hazard based on age, *APOE* genotype, sex, education, family history of dementia, vascular risk, subjective memory concerns, and baseline cognitive performance. The four cohorts varied considerably in age, education, ethnicity/race, and *APOE*-e4 allele frequency. Overall, cumulative incidence was uniformly higher in NACC than in the population-based cohorts. Among *APOE*-e4/e4 individuals, 5-y cumulative incidence was as follows: in the 60–64-y age stratum, it ranged from 0% to 5.88% in the three population-based cohorts versus 23.06% in NACC; in the 65–69-y age stratum, from 9.42% to 10.39% versus 34.62%; and in the 70–75-y age stratum, from 18.64% to 33.33% versus 38.34%. Five-year incidence of dementia was negligible except for *APOE*-e4/e4 individuals and those over 70 y. Lifetime incidence (to age 80–85 y) of MCI or dementia for the *APOE*-e4/e4 individuals in the long-term Framingham and Rotterdam cohorts was 34.69%–38.45% at age 60–64 y, 30.76%–40.26% at 65–69 y, and 33.3%–35.17% at 70–75 y. Confidence limits for these estimates are often wide, particularly for *APOE*-e4/e4 individuals and for the dementia outcome at 5 y. In regression models, *APOE-*e4 dose and age both consistently increased risk, as did lower education, subjective memory concerns, poorer baseline cognitive performance, and family history of dementia. We discuss several limitations of the study, including the small numbers of *APOE*-e4/e4 individuals, missing data and differential dropout, limited ethnic and racial diversity, and differences in definitions of exposure and outcome variables.

**Conclusions:**

Estimates of the absolute risk of MCI or dementia, particularly over short time intervals, are sensitive to sampling and a variety of methodological factors. Nonetheless, such estimates were fairly consistent across the population-based cohorts, and lower than those from a convenience cohort and those estimated in prior studies—with implications for informed consent and design for clinical trials targeting high-risk individuals.

## Abstract 2

In an analysis of four longitudinal cohort studies, Deborah Blacker and colleagues estimate the risk of mild cognitive impairment and dementia by APOE-status.

## Author summary

**Why was this study done?:**

- Having one copy of the e4 variant of the *APOE* gene (*APOE*-e4) is associated with increased risk and earlier onset age of Alzheimer disease dementia, and the 1%–2% of the population who carry two copies of this variant (*APOE*-e4/e4) are at especially increased risk.
- For this reason, individuals with *APOE*-e4/e4 are being targeted for special prevention strategies.
- Accurate risk estimates allow potential participants in prevention trials to compare the risk of developing Alzheimer disease dementia to any risk associated with the preventive intervention itself.
- Such estimates also help those planning trials decide how many participants they need.
- The risk that an individual with *APOE*-e4/e4 will develop Alzheimer disease dementia has been reported to be as high as 50%–67%, but these estimates come from statistical modeling, not direct observation.
- Our study was designed to develop risk estimates for potential participants in the Generation Study, a prevention trial in individuals with *APOE*-e4/e4, as well as estimates for those with one or no copies of *APOE*-e4. The non-*APOE*-e4/e4 individuals are not eligible for the Generation Study, but participate in genetic counseling as part of the recruiting process, and might be eligible for future trials.

**What did the researchers do and find?:**

- We examined data from 16,844 individuals, 292 with *APOE*-e4/e4, from four different samples of cognitively normal older individuals aged 60–75 years (consistent with the eligibility criteria for the Generation Study). These individuals have been followed for an average of 4 to 18 years and assessed for the development of dementia or a less serious condition called mild cognitive impairment that sometimes leads to dementia.
- We divided each sample into six groups based on the number of copies of *APOE*-e4 (none, one, and two, i.e., *APOE*-e4/e4) and age (60–64, 65–69, and 70–75 years), and developed estimates of the risk of developing mild cognitive impairment or dementia in each group during the five years planned for the trial as well as “lifetime” risk through age 80–85 years.
- Five-year risk was highly variable across the four samples within each group; for *APOE*-e4/e4 individuals, it ranged from 0% to 23% in those entering the study at age 60–64 years, 9% to 35% in those entering at 65–69 years, and 19% to 38% in those entering at 70–75 years.
- Lifetime risk was more consistent across the two samples in which it could be estimated, and did not vary as much with age, ranging from 31% to 40% for those with *APOE*-e4/e4.
- We developed statistical models to understand the differences across the four samples. These analyses showed that, beyond age and *APOE*-e4, the following variables were associated with increased risk: less education, memory concerns or poorer cognitive screening test scores at the beginning of follow-up, and having a family history of dementia.
- Differences in these characteristics, differences in methods of recruiting and evaluating participants, and statistical fluctuation probably account for a large share of the variety in risk estimates across the samples.

**What do these findings mean?:**

- The risk of dementia in those with *APOE*-e4/e4 is somewhat lower than previously estimated.
- Short-term risk is more subject to variability due to details of how a particular study is conducted than longer-term risk.
- This information is valuable to those considering joining a prevention trial, as well as those designing such trials.

## Introduction

At present, 48 million people worldwide have dementia, and this number is projected to increase to 131 million by 2050 [1]. Consequently, prevention of Alzheimer disease, the most common cause of dementia, has become a major research focus, with several prevention trials now underway [2–7]. The feasibility of these trials will in part depend on the ability to recruit individuals at risk of developing disease during the trial period. One strategy to achieve this focuses on individuals at high genetic risk. The Alzheimer’s Prevention Initiative [8] is embarking on two clinical trials targeting cognitively unimpaired individuals at highest genetic risk for Alzheimer disease, one trial in an extended early-onset Columbian kindred carrying a fully penetrant presenilin 1 mutation, and the Generation Study (NCT02565511), a trial in individuals aged 60–75 y who carry two copies of the Alzheimer disease risk allele *apolipoprotein E* epsilon 4 (*APOE*-e4). The Generation Study is a double-blind, randomized, placebo-controlled clinical trial of two different anti-amyloid agents in approximately 1,300 participants. Recruitment is through several sources, notably in the United States through the GeneMatch [9] Alzheimer disease prevention registry (NCT02564692). High-volume recruitment efforts are required because the *APOE*-e4/e4 genotype occurs in approximately 1%–2% of the general population, so thousands of individuals must be screened to identify eligible participants. An assessment of absolute risk among trial-eligible individuals in a meaningful time frame is essential for the informed consent process in the trial, as well as trial design. However, although numerous studies [10–14] document that *APOE*-e4 increases the *relative* risk of Alzheimer disease (compared to no copies of *APOE*-e4, there is a 2- to 4-fold increase in risk for one copy of *APOE*-e4, and an 8-to 15-fold increase for two copies), its effect on *absolute* risk is less clear.

When this study was begun, available estimates of absolute risk of dementia for *APOE*-e4 carriers were largely based on models developed from relative risks observed in one population and incidence data from another, often from case–control samples. The Risk Evaluation and Education for Alzheimer’s Disease (REVEAL) study [15,16] developed risk estimates [17] based on observed absolute risks in first-degree relatives versus spouses in a family sample [18], and then applied relative risks by sex, age, and genotype from a large meta-analysis [14]. A more recent effort [19], also reported on the 23andMe website [20], applied relative risks from a recent European genome-wide association study (GWAS) sample [21] to incidence estimates from the Rochester [22] and Personnes Agées QUID (PAQUID) [23] cohorts to compute lifetime risks by *APOE* genotype. Since that time, estimates from a single convenience cohort have been published, also with high incidence rates [24].

Because the available estimates of the *APOE*-associated incidence of mild cognitive impairment (MCI) or dementia are primarily based on models of disease onset rather than prospective observations, and because *APOE* also affects longevity and risk for diseases other than dementia, we developed new estimates in population-based cohorts to better inform both trial designers and potential participants. For potential Generation Study participants, the outreach and recruitment protocol for those who do not know their *APOE* genotype includes institutional review board (IRB)–approved processes for obtaining their genotype and inviting them to a trial site for an initial disclosure visit. To ensure an appropriate disclosure setting during trial enrollment, some prospective participants without the *APOE*-e4/e4 genotype are also invited for this initial genetic disclosure visit. Our aims were to use prospective data to determine 5-y and lifetime risk of MCI or dementia by age and *APOE*-e4 dose among those as similar as possible to eligible trial participants (age 60–75 y, normal cognition) and to identify sources of heterogeneity that may account for variation in risk across populations.

## Methods

### Ethics statement

The Rotterdam Study was approved by a medical ethics committee according to the Population Study Act Rotterdam Study, executed by the Ministry of Health, Welfare and Sport of the Netherlands; written informed consent was obtained from all participants. The Framingham Heart Study was reviewed by the IRB at Boston University Medical Center, and all participants gave written informed consent. The Sacramento Area Latino Study on Aging (SALSA) was reviewed by the IRBs at the University of Michigan and at the University of California at San Francisco and at Davis, and all participants gave written informed consent. Collection of data for the National Alzheimer’s Coordinating Center (NACC) Uniform Data Set cohort was reviewed by the appropriate local IRB at each participating Alzheimer’s Disease Center, and all participants gave written informed consent; research using the NACC database was approved by the University of Washington IRB. The IRBs at Partners HealthCare in Boston and the University of Massachusetts Amherst provided additional approvals for the secondary data analysis reported here. This study is reported as per STROBE reporting guidelines (S1 Checklist).

### Cohort selection

We sought available data from longitudinal population-based cohorts based on the following attributes: recruitment and an initial cognitive evaluation at or before age 60 y (because the Generation Study is recruiting individuals aged 60–75 y, and we wanted our risk assessments to be maximally relevant to those entering the trial), ongoing surveillance for assessment of MCI and dementia, and available *APOE* genotypes. Many aging-focused cohorts (e.g., the Religious Orders Study [25] and the Cache County Study [26]) did not meet these criteria because of initial ascertainment at older ages. We also sought as broad ethnic representation as possible: we were able to include one Hispanic population with limited sample size, but no African-American cohort was available with the requisite data.

Three population-based cohorts were analyzed: the Framingham Heart Study [27], the Rotterdam Study [28], and the SALSA Study [29,30]. For comparison, we also included the NACC Uniform Data Set longitudinal convenience cohort [31] (from the multi-site Alzheimer’s Disease Center Program funded by the US National Institute on Aging) because we believed that NACC participants might resemble those volunteering for the Generation Study in terms of key demographic variables and level of research interest.

### Sample selection for the present analyses

Within each cohort, we selected participants with known *APOE* genotype who were cognitively unimpaired at the time of their first visit within the 60–75-y age window, and included all available subsequent visit information until diagnosis of MCI or dementia. For the two longer-term studies, the Framingham Heart Study and the Rotterdam Study, individuals could contribute to multiple age strata for the stratified analyses, but they were included only once in our regression analyses (see “Statistical analysis” below). *APOE* genotype was measured in 94.1% (Rotterdam Study), 68.5% (Framingham Heart Study), 76.1% (NACC), and 92.0% (SALSA) of otherwise eligible (i.e., cognitively normal in the age window of 60–75 y) cohort participants, and only these individuals were included in the current study. On average, individuals without *APOE* genotype available were slightly older, except in the Framingham Heart Study, where they were slightly younger; in all cases the mean difference between those with and without *APOE* available was less than 1 y. Those without *APOE* genotype were more likely to be female in the NACC and Rotterdam cohorts, and more likely to be male in the SALSA and Framingham cohorts, but these differences were also small—within 1%–2%, except for the Rotterdam cohort, where females were 66.1% of those without genotype and 54.8% of those with genotype.

### Ascertainment and assessment methods for each cohort

The original Framingham Heart Study cohort was recruited in 1948–1953 based on residence in Framingham, Massachusetts, for a longitudinal study of cardiovascular disease (mean age at enrollment 45 y). A cohort of offspring of the original participants and their spouses was established in 1971–1975 (mean age at enrollment 37 y). Details of study procedures have been published elsewhere [27]. Cognitive status has been monitored in the original cohort since 1975, when a comprehensive neuropsychological battery was administered, followed by neurological assessment of participants with lower cognitive test scores [32]. Since 1981, this cohort has been assessed at each examination with a Mini-Mental State Examination (MMSE), where participants were flagged for further cognitive screening if they scored below predefined cutoffs based on education and prior performance. The offspring cohort has undergone similar monitoring with serial MMSEs since 1991. Participants identified as having possible cognitive impairment based on these screening assessments (or in reports of cognitive concerns by the participant, family, treating physician, or Framingham ancillary study investigators, or through review of outside medical records) are invited to undergo additional annual neurological and neuropsychological examinations. A dementia review panel including a neurologist and a neuropsychologist reviews each case of possible cognitive decline and dementia and categorizes participants based on the best available information (from serial neurological and neuropsychological assessments, telephone interviews with caregivers, medical records, neuroimaging, and, when available, autopsy data) and assigns a diagnosis and onset date for dementia according to DSM-IV criteria and for MCI based on Petersen et al. [33] criteria. Diagnoses made prior to 2001 have been re-reviewed to update diagnostic criteria. Participants who entered the sample for the present analyses at a visit prior to MMSE administration but who were cognitively unimpaired at subsequent study visits had this designation extended back to their earlier visits. For our regression analyses, these individuals were included with the baseline visit as the first visit with MMSE administration within our age window (60–75 y).

For the Rotterdam Study, individuals over 55 y in 1990 residing in a specific district of the City of Rotterdam, the Netherlands, were invited to participate, with additional waves invited in 2000 (age >55 y) and 2005 (age >45 y). Details of study procedures have previously been published [28]. In brief, all participants were interviewed at home and examined at the study center every 4 to 5 y. Participants were routinely screened for dementia at the initial visit and follow-up examinations using a three-step protocol. Screening was done using the MMSE and the Geriatric Mental Schedule (GMS) organic level [2]. Those with MMSE < 26 or GMS organic level > 0 subsequently underwent an examination and informant interview using the Cambridge Examination for Mental Disorders of the Elderly (CAMDEX) [34]. Additionally, the total cohort was continuously monitored for dementia through computerized linkage between the study database and digitized medical records. The current sample included all participants with MMSE > 26 at the time of their first visit within the age window of interest (60–75 y). Formal assessment of MCI did not begin until 2005 in the Rotterdam Study. For the present analyses, we therefore developed a pragmatic diagnosis of MCI during follow-up, requiring a MMSE score < 26 or a drop of at least three points from the baseline visit in the 60–75-y age window, plus answering yes to a question about memory concerns.

For SALSA, participants over 60 y were sampled from six counties including census tracts with at least 5% Hispanic population in the Sacramento Valley of California in 1998–1999 and were followed approximately every 12–15 mo until 2008. Detailed methods are described elsewhere [29,35]. In brief, dementia assessment included screening with both the Modified Mini-Mental State Examination (3MS) [36] and a word list learning task from a standard battery [30]. Those scoring below the 20th percentile (using age-, education-, sex-, and language-adjusted norms) on either test (or for follow-up visits, dropping three points in word list learning) were further evaluated using the Informant Questionnaire on Cognitive Decline in the Elderly (IQCODE) [37,38] and, if this gave additional support for decline, were evaluated by a neurologist and categorized as cognitively unimpaired, memory-impaired (based on testing alone, without IQCODE corroboration), cognitively impaired not demented (CIND) [39], or having dementia. Given the requirement for both a cognitive testing abnormality and confirmation from an informant, CIND was treated as equivalent to MCI [35].

Participants in the NACC cohort were volunteers ascertained from various sources at 34 Alzheimer’s Disease Centers in the United States. We used the March 2016 data freeze for the present analyses, so these data reflect study visits between September 2005 and March 2016. The participants were evaluated according to the Uniform Data Set protocol [40], with each participant and a collateral informant interviewed by the study clinician to rate the Clinical Dementia Rating (CDR) [41] and with the administration of a battery of neuropsychological tests [42]. A diagnosis was made at each visit by the study clinician following standard criteria [40], but there were no study-wide standardized cutoffs on the CDR, MMSE, or other neuropsychological tests. Follow-up visits were conducted approximately annually.

### Definition of predictor variables

Education was reported in years for the SALSA and NACC cohorts and in categories of less than high school, high school, some college, or college graduation for the Rotterdam and Framingham cohorts. Education data for the SALSA and NACC cohorts were translated into these categories as follows: <12 y, less than high school; 12 y, high school; 13–15 y, some college; and ≥16 y, college graduation.

To assess cognitive performance across cohorts, we used the cognitive screening test available for each site (MMSE for the Rotterdam Study, the Framingham Heart Study, and NACC, and 3MS for SALSA). To enable comparisons of relative performance within each cohort, we standardized based on the test score at the baseline visit (in the age 60–75-y age window) within each cohort, centering the raw scores around their sample mean and then dividing the centered scores by their standard deviation.

Memory concerns at NACC were based on a global clinician-rated variable asking whether the participant believed that he or she had a problem with memory. Memory concerns in the Rotterdam Study were based on three questionnaire items asking (1) whether the participant was worried about his or her memory, (2) whether the participant ever lost track of what he or she was doing in the midst of an activity, and (3) whether the participant experienced word-finding difficulties. A positive answer to any of these questions qualified as memory concerns.

Family history of dementia was defined as having at least one parent with dementia for the Rotterdam Study, and at least one first-degree relative with dementia for NACC.

For all cohorts, vascular risk was defined as follows. We obtained the sum of major vascular risk factors measured in each cohort: coronary artery disease or angina or stroke, hypertension, high cholesterol, diabetes, atrial fibrillation, and current smoking. After reviewing the distribution of the count of these risk factors (range 0–6), we categorized the participants as high risk (3–6 risk factors), moderate risk (1–2 risk factors), and low risk (0 risk factors) to provide a reasonable distribution across the three levels. We considered using the Framingham Stroke Risk Profile [43] or similar cardiovascular risk scores [44,45]. However, these were designed to predict risk within a specified time frame and thus had substantial age components, which complicated analyses in our regression models that already included age.

### Statistical analysis

We performed all analyses first for MCI or dementia (“MCI/dementia”), then for dementia alone. For the purposes of this trial, the MCI/dementia outcome was critically relevant, in that incident dementia was unlikely during the trial period, while there was tangible risk for MCI. Analyses for dementia only were performed as well because dementia is a more robust outcome than MCI.

We estimated 5-y and “lifetime” (i.e., to age 80–85 y) cumulative incidence by *APOE*-e4 dose and 5-y baseline age stratum (age 60–64, 65–69, 70–75 y). We chose three age strata as a tradeoff between addressing the steeply changing risk with age and not overly subdividing the limited numbers of *APOE*-e4/e4 homozygote individuals, which left the number of *APOE*-e4/e4 individuals per age stratum too small for stable estimates in the SALSA cohort. These age strata were determined specifically as follows, based on the date of the baseline visit (within the 60–75-y age window): 60–64 y, 60 ≤ age < 65; 65–69 y, 65 ≤ age < 70; and 70–75, 70 ≤ age ≤ 75. We considered further stratification on sex, but the sample size did not support such stratification.

For the stratified analyses of the two longer-term studies, the Framingham Heart Study and the Rotterdam Study, individuals could contribute to multiple baseline age strata; we used the first visit within each age window as the baseline in these analyses. Lifetime estimates were computed as 20-y cumulative incidence for the age 60–64-y stratum, as 15-y for the 65–69-y stratum, and as 10-y for the 70–75-y stratum; these estimates were computed only for the two longer-term cohorts to minimize extrapolation.

Stratified cumulative incidence curves by age stratum and *APOE*-e4 dose were estimated, adjusting for loss to follow-up other than death and for the competing risk of mortality [46]; loss to follow-up other than death is treated as censored. In the presence of competing risks, the naïve Kaplan–Meier estimator, which treats failure from competing causes as censored observations, overestimates the cumulative incidence of the event of interest [47]. We used the “cmprsk” package in R software [48] to estimate the cumulative incidence for each age stratum by *APOE*-e4 dose stratum. Following the suggestion of Lin [49], we used the transformation log[−log(1 − *x*)] to construct the confidence interval for cumulative incidence. The transformation not only ensures that the boundaries of cumulative incidence are contained in [0,1], but also improves the coverage accuracy [49].

We used the same competing risks analytic framework to assess the effects of age and *APOE*-e4 dose plus additional covariates on the cumulative incidence of MCI/dementia and of dementia alone in order to inform personalized risk assessment and to understand differences across the cohorts. We used subdistribution hazard regression models [50] because they directly link the regression coefficients with the cumulative incidence function (in contrast to cause-specific hazards regression [51], where the direct link cannot be made [52]; in preliminary analyses we also fit these models, and results were very similar). These analyses were also performed using the “cmprsk” package in R software [48].

For each cohort and for each outcome, we first fit univariable models for baseline age, sex, *APOE*-e4 dose, education, standardized cognitive screening test score, subjective memory concerns, family history of dementia, and vascular risk score. Then, we ran simple multivariable models for each outcome including only *APOE*-e4 dose and demographic factors (age, sex, and education). Last, we ran larger multivariable models also including standardized cognitive screening score plus subjective memory concerns and family history of dementia if available for the cohort. The vascular risk score was not included in the full model because findings were inconsistent and primarily null in the univariable models, whether we used our low/moderate/high vascular risk levels described above or the Framingham Stroke Risk Profile.

Missing data on covariates was generally minimal, <2% for all covariates in all cohorts except family history in the Rotterdam Study (11.5%), vascular risk in SALSA (8.9%), and education in the Framingham Heart Study (3.3%). As these figures were small, participants with missing data were simply omitted from regression analyses in which the relevant missing variable was included.

For the Rotterdam Study, the exact date of dementia diagnosis was used if available; otherwise, the midpoint of the interval between visits was used as the onset time of MCI or dementia at a study visit (conducted at 4-y intervals) for both cumulative incidence estimates and subdistribution hazard regression. In addition, as a sensitivity analysis, we repeated our survival curves and regression models treating the onset of MCI or dementia as interval censored in addition to adjusting for competing risk, using the “MIICD” package in R software to estimate the cumulative incidence, and results were extremely similar except for somewhat larger confidence intervals.

Unlike in the stratified analyses, in the regression analyses, each participant was used only once. Typically, the baseline visit for the regression analyses was the first visit within the eligible age window of 60–75 y. For the Framingham Heart Study, MMSE was not available at visits prior to 1981 (as described above). Thus, for the regression analyses, we reset the baseline visit as the first visit at which MMSE was available. This had the additional benefit of increasing the range of baseline ages within the cohort.

Meta-analyses were conducted for the 5-y cumulative incidence estimates for all four cohorts and then for only the three population-based cohorts. Meta-analyses could not be conducted for the lifetime estimates because they were computed for only two cohorts. As there was considerable heterogeneity among the studies, a random-effects meta-analysis based on the DerSimonian–Laird method [53] was used. This analysis was performed using the “metafor” package in R software.

Because the primary goal was estimating cumulative incidence and understanding differences across cohorts and individuals rather than hypothesis testing, these analyses are reported with confidence intervals rather than statistical significance, and no adjustments are made for multiple comparisons.

The study was planned in summer of 2014 and conducted through fall of 2016. The original analysis plan, developed in the initial months of the study, specified using multiple cohort studies, including NACC as well as three population-based cohorts; stratified analyses in the three age and *APOE*-e4 dose groups; and regression models including demographic factors, cardiovascular risk, and baseline cognitive performance or symptoms, with survival models accounting for competing risk of death. The idea was that the stratified curves would provide general estimates, and the regression models more individualized estimates (and in any event insight into anticipated differences across cohorts). As noted above, after initial exploration, we decided to use specifically subdistribution hazard regression rather than cause-specific hazard regression models because these offer greater interpretability in the context of risk prediction. In preparation for our first presentation of the findings to the Generation Study team in April 2015, we also decided to make a table of estimated 5-y cumulative incidence to allow easier comparison across the studies. After this initial meeting, we also added the lifetime risk estimate, which we thought would be informative for potential participants. We performed the initial regression models separately by cohort in order to get an idea of how best to present the data given somewhat different variables available in each cohort, and then settled on a final analysis plan in spring 2016 that included univariable and two nested models as consistent as possible across the four cohorts. The NACC dataset was acquired in summer 2014 (the June 2014 data freeze) and then updated in summer 2016 (the March 2016 data freeze). The SALSA dataset was acquired in fall 2014, and the Framingham Heart Study dataset in spring 2015. Rotterdam Study analyses were conducted by Rotterdam Study investigators (F. J. W. and M. A. I.) beginning in spring 2015, in close collaboration with the rest of the group and sharing R code used with the other cohorts to ensure consistency.

## Results

### Composition of the cohorts

Table 1 presents the composition of the four cohorts. The cohorts differed considerably in size and duration of follow-up, with SALSA much smaller than the other cohorts, and long-term follow-up available only in the Framingham Heart Study and Rotterdam Study. Other substantial differences were seen in educational attainment, with mean years ranging from less than 8 y in SALSA to nearly 16 y in NACC, and sex, with 33.6% men in NACC compared to 42%–45% in the three population-based cohorts. The four cohorts also differed markedly in *APOE*-e4 allele frequency, ranging from 7.5% in SALSA to 17.8% in NACC. NACC also had a 58.3% fraction with a family history of dementia, compared to 18.6% in the Rotterdam Study, the only other site that assessed it.

**Table 1:** Demographic and clinical characteristics of participants in our samples from the National Alzheimer’s Coordinating Center, the Rotterdam Study, the Framingham Heart Study, and the Sacramento Area Latino Study on Aging.

Characteristic	Cohort			
	NACC (*n* = 5,073)	RS (*n* = 6,399)	FHS (*n* = 4,078)	SALSA (*n* = 1,294)
**Remaining at 5 y of follow-up**	1,865 (36.7%)	5,592 (87.4%)	3,911 (95.9%)	976 (75.5%)
**Length of follow-up (years)**	3.96 (2.97)	12.64 (6.14)	17.59 (9.09)	6.50 (2.52)
**MCI or dementia cases**	602 (11.9%)	1,301 (20.3%)	826 (20.3%)	111 (8.6%)
**Dementia cases**	55 (1.1%)	782 (12.2%)	658 (16.1%)	49 (3.8%)
**Male sex**	1,707 (33.6%)	2,893 (45.2%)	1,762 (43.2%)	538 (41.6%)
**Age (years) at baseline visit**	68.7 (4.30)	65.4 (4.18)*	62.0 (1.71)*	67.8 (4.42)
**Education (years)**^#^	15.79 (2.99)	12.94 (N/A)	13.20 (N/A)	7.72 (5.42)
**Education level**				
Less than high school	140 (2.8%)	728 (11.4%)	622 (15.3%)	835 (64.5%)
High school	720 (14.2%)	2,773 (43.3%)	1,330 (32.6%)	201 (15.5%)
Some college	815 (16.1%)	1,965 (30.7%)	1,004 (24.6%)	126 (9.7%)
College graduation	3,379 (66.6%)	871 (13.6%)	994 (24.4%)	125 (9.7%)
***APOE*-e4 allele frequency**	17.8%	15.0%	11.7%	7.5%
**Genotype**				
e2/e2, e2/e3, e3/e3	3,431 (67.6%)	4,598 (71.9%)	3,166 (77.6%)	1,112 (85.9%)
e2/e4, e3/e4	1,484 (29.3%)	1,645 (25.7%)	845 (20.7%)	171 (13.2%)
e4/e4	158 (3.1%)	156 (2.4%)	67 (1.6%)	11 (0.9%)
**Family history of dementia**	2,957 (58.3%)	1,191 (18.6%)	N/A^†^	N/A^†^
**Cognitive screening test score (MMSE or 3MS)**^**‡**^	29.0 (1.3)	28.5 (1.0)	28.8 (1.4)	86.7 (11.2)
**Subjective memory concerns**	1,262 (24.9%)	2,759 (43.1%)	N/A^§^	N/A^§^
**Vascular risk score**^**$**^	1.32 (1.06)	1.82 (0.85)	1.38 (0.94)	2.13 (1.16)
**Vascular risk level**				
Low (0)	1,264 (24.9%)	263 (4.1%)	702 (17.2%)	70 (5.41%)
Moderate (1/2)	3,096 (61.0%)	4,916 (76.8%)	2,923 (71.7%)	737 (57.0%)
High (3/4/5/6)	713 (14.1%)	1,120 (19.1%)	453 (11.1%)	439 (33.9%)

Data are given as *n* (percent), mean (standard deviation), or percent.

*For the Framingham Study and the Rotterdam Study, this refers to the age in years of the first study visit within the age 60–75-y window included in the present analyses. In the regression analyses, mean age in the Framingham Heart Study was 65.39 (standard deviation 3.87) because we used the first visit with available MMSE as the baseline (see text).

^#^In the Framingham Heart Study and the Rotterdam Study, the education variable was recorded as categories. To facilitate comparisons with other samples, the mean was estimated by considering less than high school as 10 y, high school as 12 y, some college as 14 y, and college graduate as 16 y. Education data for the NACC and SALSA cohorts were translated into the categories as follows: <12 y, less than high school; 12 y, high school; 13–15 y, some college; and ≥16 y, college graduation.

^†^Family history of dementia was not available in the SALSA and Framingham Heart Study cohorts.

^‡^MMSE (range [0, 30], with typical dementia cutoff at ~24); for SALSA, the 3MS was used (range [0,100], with typical dementia cutoff at ~77).

^§^Subjective memory concerns was not available in the SALSA and Framingham cohorts.

^$^Vascular risk score was calculated based on a count of standard risk factors, see text for details.

3MS, Modified Mini-Mental State Examination; FHS, Framingham Heart Study; MCI, mild cognitive impairment; MMSE, Mini-Mental State Examination; N/A, not available; NACC, National Alzheimer’s Coordinating Center; RS, Rotterdam Study; SALSA, Sacramento Area Latino Study on Aging.

### Stratified cumulative incidence estimates

**Fig 1:**
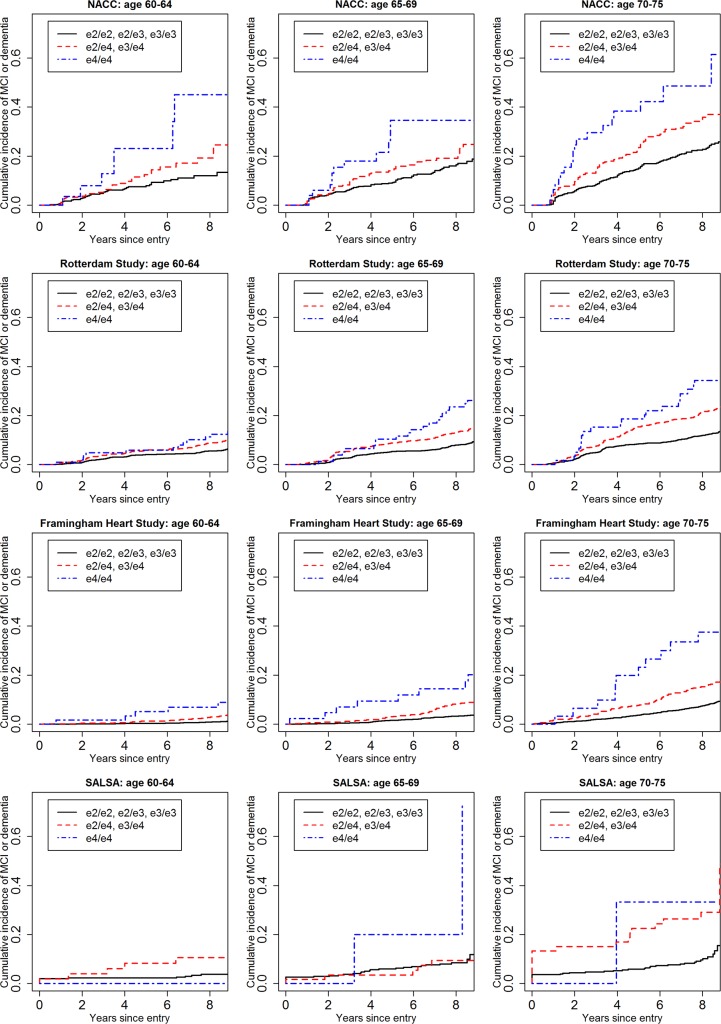
Cumulative incidence curves, adjusting for competing risk of mortality, for mild cognitive impairment or dementia by baseline age and *APOE*-e4 dose. Note that the strata shown are not independent for the Rotterdam and Framingham cohorts (see text). MCI, mild cognitive impairment; NACC, National Alzheimer’s Coordinating Center; RS, Rotterdam Study; SALSA, Sacramento Area Latino Study on Aging.

**Fig 2:**
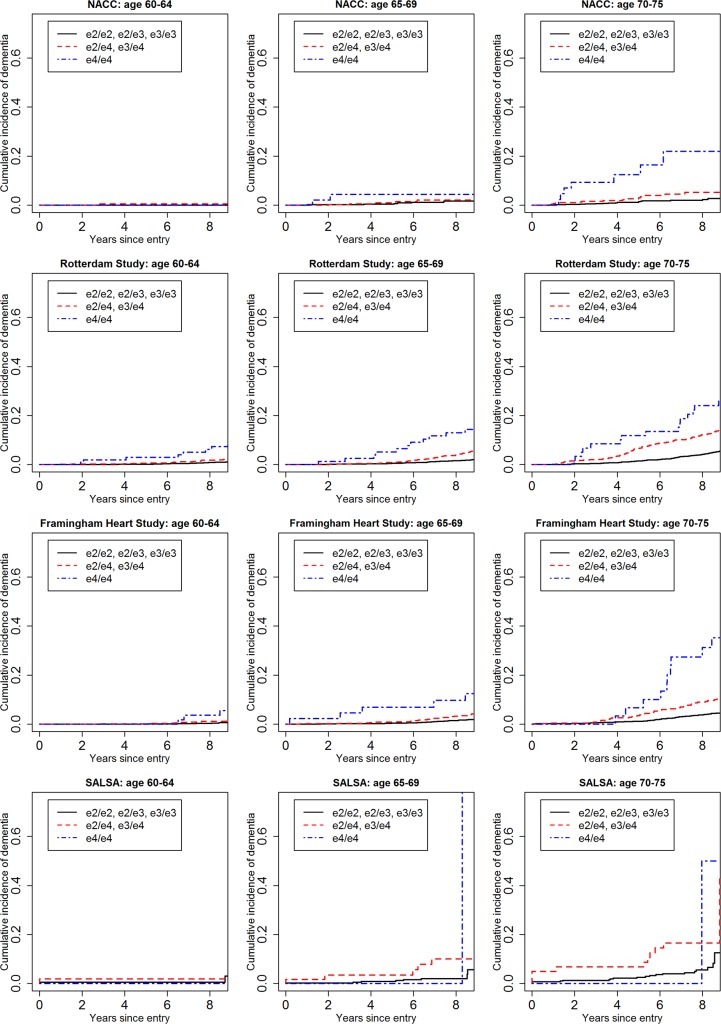
Cumulative incidence curves, adjusting for competing risk of mortality, for dementia by baseline age and *APOE*-e4 dose. Note that the strata shown are not independent for the Rotterdam and Framingham cohorts (see text). NACC, National Alzheimer’s Coordinating Center; RS, Rotterdam Study; SALSA, Sacramento Area Latino Study on Aging.

**Fig 3:**
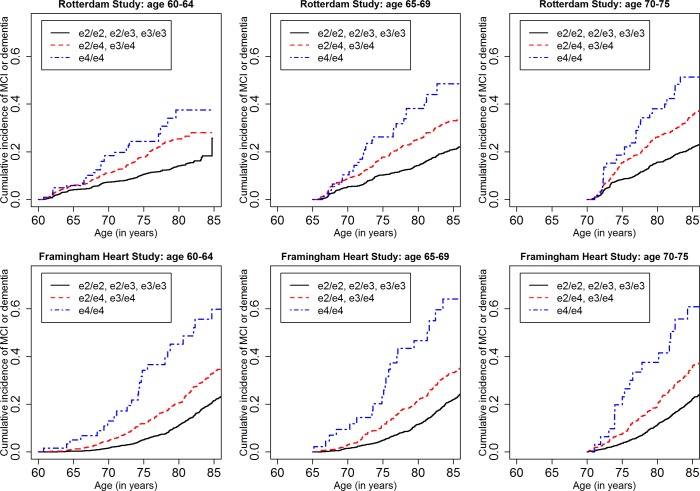
Lifetime (to age 80–85 y) cumulative incidence curves, adjusting for competing risk of mortality, for mild cognitive impairment or dementia by baseline age and *APOE*-e4 dose. Note that the strata shown are not independent (see text). MCI, mild cognitive impairment.

**Fig 4:**
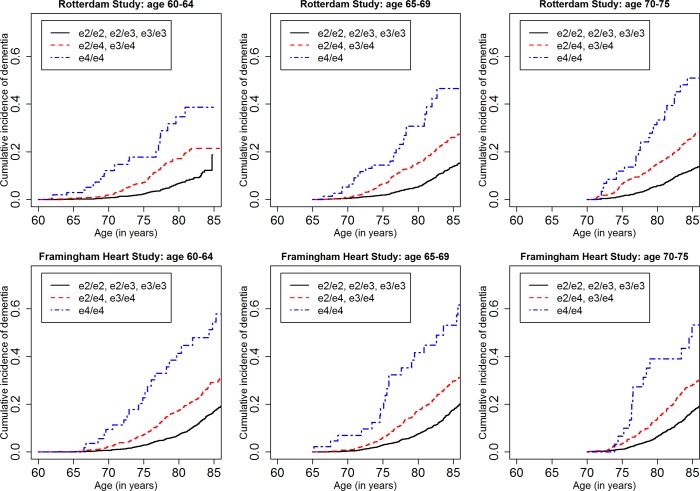
Lifetime (to age 80–85 y) cumulative incidence curves, adjusting for competing risk of mortality, for dementia by baseline age and *APOE*-e4 dose. Note that the strata shown are not independent (see text).

Fig 1 shows the cumulative incidence of MCI/dementia stratified by baseline age group and *APOE*-e4 dose; Fig 2 shows the corresponding curves for dementia alone. These figures show 8.5 y of follow-up for all four cohorts on the same scale, to facilitate comparison. Figs 3 and 4 show lifetime (to age 80–85 y) cumulative incidence curves for MCI/dementia and dementia alone for the two longer-term cohorts.

Table 2 shows the 5-y cumulative incidence of MCI/dementia for all four cohorts, and Table 3 the lifetime (to age 80–85 y) cumulative incidence across the two longer-term cohorts; Tables 4 and 5 show the corresponding data for dementia alone.

**Table 2:** Five-year cumulative incidence of mild cognitive impairment/dementia by baseline age and *APOE*-e4 dose.

Age (years) and *APOE*	Cohort								Meta-analysis (RS, FHS, SALSA): 5-y cumulative incidence, percent (95% CI)	Meta-analysis (four cohorts): 5-y cumulative incidence, percent (95% CI)
	NACC		RS*		FHS*		SALSA			
	*n*	5-y cumulative incidence, percent (95% CI)	*n*	5-y cumulative incidence, percent (95% CI)	*n*	5-y cumulative incidence, percent (95% CI)	*n*	5-y cumulative incidence, percent (95% CI)		
**Age 60–64**										
0 e4	740	7.94 (5.65, 11.10)	2,625	4.08 (3.39, 4.91)	2,955	0.31 (0.16, 0.59)	352	2.29 (1.15, 4.53)	1.46 (0.31, 6.72)	2.29 (0.84, 6.21)
1 e4	322	12.38 (8.14, 18.61)	928	5.72 (4.40, 7.42)	795	1.28 (0.69, 2.37)	51	8.28 (3.15, 20.80)	3.86 (1.32, 11.03)	5.27 (2.24, 12.13)
2 e4	36	23.06 (9.94, 48.12)	102	5.88 (2.68, 12.67)	62	5.07 (1.65, 15.04)	3	0.00 (0.00, 0.00)	5.60 (2.94, 10.53)	9.10 (3.38, 23.23)
**Age 65–69**										
0 e4	1,172	9.19 (7.28, 11.57)	2,492	5.38 (4.56, 6.34)	2,430	1.52 (1.10, 2.10)	377	5.97 (3.93, 9.03)	3.63 (1.34, 9.62)	4.60 (1.85, 11.21)
1 e4	585	14.40 (11.08, 18.61)	906	8.84 (7.16, 10.89)	646	3.02 (1.94, 4.70)	60	3.45 (0.86, 13.28)	4.84 (1.96, 11.67)	6.75 (3.47, 12.97)
2 e4	65	34.62 (20.15, 55.18)	77	10.39 (5.31, 19.79)	44	9.42 (3.60, 23.45)	5	20.00 (2.45, 86.54)	10.52 (6.19, 17.57)	16.36 (7.27, 34.51)
**Age 70–75**										
0 e4	1,519	15.16 (13.03, 17.61)	2,212	8.42 (7.33, 9.65)	1,888	3.71 (2.93, 4.68)	383	5.88 (3.91, 8.80)	5.71 (3.19, 10.13)	7.37 (4.06, 13.19)
1 e4	577	23.56 (19.48, 28.34)	739	15.45 (13.03, 18.27)	464	7.71 (5.60, 10.59)	60	22.49 (13.63, 35.77)	13.66 (7.88, 23.11)	15.86 (10.02, 24.61)
2 e4	57	38.34 (25.09, 55.48)	59	18.64 (10.73, 31.28)	32	23.16 (11.61, 43.01)	3	33.33 (2.68, 99.76)	20.58 (13.51, 30.65)	26.70 (17.50, 39.45)

*For the Framingham Heart Study and the Rotterdam Study, individuals could contribute to multiple baseline age strata. In the meta-analysis, cumulative incidence 0.00 (0.00, 0.00) was not used.

FHS, Framingham Heart Study; NACC, National Alzheimer’s Coordinating Center; RS, Rotterdam Study; SALSA, Sacramento Area Latino Study on Aging.

**Table 3:** Lifetime (to age 80–85 y) cumulative incidence of mild cognitive impairment/dementia by baseline age and *APOE*-e4 dose.

Age (years) and APOE	Cohort			
	RS*		FHS*	
	*n*	Lifetime cumulative incidence, percent (95% CI)	*n*	Lifetime cumulative incidence, percent (95% CI)
**Age 60–64**				
0 e4	2,625	14.05 (12.50, 15.78)	2,955	11.94 (10.56, 13.49)
1 e4	928	25.37 (21.96, 29.21)	795	22.07 (18.69, 25.94)
2 e4	102	37.47 (25.11, 53.34)	62	45.15 (31.32, 61.70)
**Age 65–69**				
0 e4	2,492	14.23 (12.83, 15.76)	2,430	12.22 (10.80, 13.81)
1 e4	906	25.44 (22.50, 28.69)	646	23.33 (19.76, 27.44)
2 e4	77	38.09 (27.25, 51.46)	44	46.66 (31.56, 64.65)
**Age 70–75**				
0 e4	2,212	15.57 (14.11, 17.16)	1,888	11.94 (10.43, 13.64)
1 e4	739	26.14 (23.10, 29.51)	464	21.35 (17.58, 25.80)
2 e4	59	38.02 (26.76, 52.04)	32	37.55 (22.42, 58.23)

For age 60–64 y, lifetime cumulative incidence is estimated as 20-y cumulative incidence. For age 65–69 y, lifetime cumulative incidence is estimated as 15-y cumulative incidence. For age 70–75 y, lifetime cumulative incidence is estimated as 10-y cumulative incidence.

*Individuals could contribute to multiple baseline age strata.

FHS, Framingham Heart Study; RS, Rotterdam Study.

**Table 4:** Five-year cumulative incidence of dementia by baseline age and *APOE*-e4 dose.

Age (years) and *APOE*	Cohort								Meta-analysis (RS, FHS, SALSA): 5-y cumulative incidence, percent (95% CI)	Meta-analysis (four cohorts): 5-y cumulative incidence, percent (95% CI)
	NACC		RS*		FHS*		SALSA			
	*n*	5-y cumulative incidence, percent (95% CI)	*n*	5-y cumulative incidence, percent (95% CI)	*n*	5-y cumulative incidence, percent (95% CI)	*n*	5-y cumulative incidence, percent (95% CI)		
**Age 60–64**										
0 e4	740	0.00 (0.00, 0.00)	2,625	0.15 (0.06, 0.41)	2,955	0.03 (0.00, 0.25)	352	0.57 (0.14, 2.26)	0.16 (0.04, 0.62)	0.16 (0.04, 0.62)
1 e4	322	0.54 (0.08, 3.79)	928	0.54 (0.22, 1.29)	795	0.00 (0.00,0.00)	51	1.96 (0.27, 13.36)	0.75 (0.25, 2.26)	0.65 (0.31, 1.36)
2 e4	36	0.00 (0.00, 0.00)	102	2.94 (0.95, 8.89)	62	0.00 (0.00,0.00)	3	0.00 (0.00, 0.00)	2.94 (0.95, 8.89)	2.94 (0.95, 8.89)
**Age 65–69**										
0 e4	1,172	0.38 (0.12, 1.23)	2,492	0.52 (0.30, 0.90)	2,430	0.25 (0.11, 0.56)	377	0.90 (0.29, 2.79)	0.46 (0.25, 0.86)	0.45 (0.28, 0.72)
1 e4	585	0.95 (0.30, 2.97)	906	0.88 (0.44, 1.76)	646	0.63 (0.24, 1.68)	60	3.45 (0.86, 13.28)	1.07 (0.48, 2.38)	1.00 (0.56, 1.78)
2 e4	65	4.36 (1.09, 16.60)	77	5.19 (1.97, 13.33)	44	4.76 (1.19, 18.00)	5	0.00 (0.00, 0.00)	5.05 (2.28, 10.96)	4.87 (2.45, 9.56)
**Age 70–75**										
0 e4	1,519	1.41 (0.81, 2.46)	2,212	1.45 (1.03, 2.04)	1,888	0.98 (0.62, 1.55)	383	2.60 (1.35, 4.95)	1.47 (0.93, 2.34)	1.44 (1.03, 2.02)
1 e4	577	3.02 (1.64, 5.55)	739	6.51 (4.94, 8.54)	464	3.09 (1.84, 5.17)	60	6.79 (2.58, 17.24)	5.06 (2.92, 8.69)	4.47 (2.75, 7.22)
2 e4	57	12.43 (5.25, 27.87)	59	11.86 (5.80, 23.43)	32	6.67 (1.67, 24.62)	3	0.00 (0.00, 0.00)	10.47 (5.55, 19.28)	11.12 (6.68, 18.20)

*For the Framingham Heart Study and the Rotterdam Study, individuals could contribute to multiple baseline age strata. In meta-analysis, cumulative incidence 0.00 (0.00, 0.00) was not used.

FHS, Framingham Heart Study; NACC, National Alzheimer’s Coordinating Center; RS, Rotterdam Study; SALSA, Sacramento Area Latino Study on Aging.

**Table 5:** Lifetime (to age 80–85 y) cumulative incidence of dementia by baseline age and *APOE*-e4 dose.

Age (years) and APOE	Cohort			
	RS*		FHS*	
	*n*	“Lifetime” cumulative incidence, percent (95% CI)	*n*	“Lifetime” cumulative incidence, percent (95% CI)
**Age 60–64**				
0 e4	2,625	6.83 (5.59, 8.33)	2,955	6.22 (5.23, 7.40)
1 e4	928	17.18 (14.06, 20.90)	795	15.93 (12.98, 19.47)
2 e4	102	34.69 (22.75, 50.49)	62	38.49 (25.50, 55.17)
**Age 65–69**				
0 e4	2,492	5.26 (4.37, 6.34)	2,430	6.59 (5.53, 7.84)
1 e4	906	15.36 (12.93, 18.20)	646	16.22 (13.16, 19.92)
2 e4	77	30.76 (20.74, 44.09)	44	40.26 (25.75, 59.00)
**Age 70–75**				
0 e4	2,212	5.76 (4.86, 6.83)	1,888	5.65 (4.61, 6.91)
1 e4	739	14.77 (12.38, 17.58)	464	13.94 (10.82, 17.86)
2 e4	59	33.30 (22.54, 47.37)	32	35.17 (20.32, 56.27)

For age 60–64 y, lifetime cumulative incidence is estimated as 20-y cumulative incidence. For age 65–69 y, lifetime cumulative incidence is estimated as 15-y cumulative incidence. For age 70–75 y, lifetime cumulative incidence is estimated as 10-y cumulative incidence.

*Individuals could contribute to multiple baseline age strata.

FHS, Framingham Heart Study; RS, Rotterdam Study.

Overall, within each cohort, risk of MCI/dementia increased with increasing age and *APOE*-e4 dose. However, absolute risks differed substantially across the cohorts, particularly between NACC and the population-based cohorts. Especially for the MCI/dementia outcome, the NACC cohort typically had higher risk for any genotype at any age. Differences among the population-based cohorts were smaller, particularly for longer-term follow-up and the dementia outcome.

Five-year cumulative incidence of MCI/dementia was low in the youngest age stratum, particularly in the cohort studies, although somewhat higher for *APOE*-e4-positive individuals, especially homozygote individuals (23% in NACC and 5%–6% in Framingham and Rotterdam). Five-year incidence of MCI/dementia was higher in the highest age stratum, particularly among *APOE*-e4/e4 homozygote individuals (38% in NACC and 18%–23% in Framingham and Rotterdam). Five-year incidence of dementia alone was negligible at younger ages, even in *APOE*-e4/e4 homozygote individuals, but rose among older individuals, particularly among those with *APOE*-e4/e4 (12% in NACC and 7%–12% in Framingham and Rotterdam). The meta-analyses of the 5-y cumulative incidence estimates for the MCI/dementia outcome (Table 2) showed consistent increases in incidence by gene dose within age strata and by age stratum within gene dose, and were higher when the NACC estimates were included. These pooled estimates ranged from a low of 1.46% for individuals aged 60–64 y with no copies of *APOE*-e4 in just the population-based cohorts to a high of 26.70% for individuals aged 70–75 y with two copies of *APOE*-e4/e4 in all four cohorts.

Estimated only for the Rotterdam Study and the Framingham Heart Study, lifetime incidence, whether for MCI/dementia or for dementia alone, was consistent in the two cohorts (it was also consistent across age strata, but it should be noted that the strata are not independent and that older age strata included individuals who survived and did not experience the outcome in earlier strata). Lifetime incidence rose consistently with *APOE*-e4 dose: for MCI/dementia (Table 3), it ranged from 11.94%–15.57% for those with no copies of *APOE*-e4 to 37.47%–46.66% for *APOE*-e4/e4 homozygote individuals; for dementia alone (Table 5), it ranged from 5.26%–6.83% for no copies to 30.76%–40.26 for homozygote individuals.

### Subdistribution hazard regression analyses

Results of the subdistribution hazard regression analysis are presented in S1 Appendix Tables A and B (univariable analyses), S1 Appendix Tables C and D (multivariable analyses modeling *APOE*-e4 dose and demographics), and S1 Appendix Tables E and F (additionally including family history of dementia and cognitive variables). Overall, the regression results were fairly consistent across the four cohorts, even in the small SALSA cohort, and considerably more consistent than the cumulative incidence results.

The univariable results (S1 Appendix Table A for MCI/dementia and S1 Appendix Table B for dementia) were fairly consistent across the two outcomes (although for some variables in some cohorts the hazard ratios [HRs] were somewhat higher for dementia alone), so we provide details in the text for the MCI/dementia outcome only. There was substantially higher risk of MCI/dementia with increasing age (HR 1.08–1.16 per year of age), increasing *APOE*-e4 dose (for one copy, HR 1.51–2.23; for two copies, HR 2.63–3.57), and lower education (HR 1.41–1.86 for less than high school compared to high school). Family history of dementia also had a nominally significant effect in both cohorts in which it was measured (HR 1.16–1.27). On the other hand, male sex, which was protective in the population-based cohorts (although only nominally significantly so in the Rotterdam cohort, HR 0.83–0.90), carried risk in NACC (HR 1.36). Subjective memory concerns carried risk in both cohorts that assessed them (HR 1.71–2.62). Higher standardized baseline cognitive screening test score (MMSE or 3MS) was consistently protective across all cohorts for both outcomes (HR 0.58–0.80 per standard deviation above the mean), except for the MCI/dementia outcome in the Rotterdam cohort. Vascular risk score had a variable and generally nonsignificant effect across all four cohorts.

The simple multivariable models including *APOE* and demographic factors (S1 Appendix Table C for MCI/dementia and S1 Appendix Table D for dementia alone) did not appreciably change the results for age and *APOE*-e4 dose, although there was some attenuation of associations for sex and education. In the more complex model (S1 Appendix Tables E and F), again the picture was similar, with attenuation for sex and education. It is noteworthy that standardized cognitive screening test score and subjective memory concerns (where available) generally showed substantial, nominally significant hazard ratios, even controlling for education, and that (where available) family history of dementia, even when controlling for *APOE*-e4 dose, also had an impact.

## Discussion

### Overall findings

Of 16,844 participants included from all four cohorts, 392 (2.3%) had the *APOE-*e4/e4 genotype, highlighting its low prevalence. Nonetheless, the expected age- and *APOE*-e4-dose-related increases in cumulative incidence and relative hazard in the regression models are readily apparent, even to some extent in the very small SALSA cohort. However, the striking differences in estimated cumulative incidence, particularly for the MCI/dementia outcome, between the population-based cohort studies and the highly ascertained NACC cohort (see below) suggest that overall *APOE*-e4-associated incidence is somewhat lower than the modeled findings previously available in the literature. Comparing 5-y cumulative incidence from the meta-analyses of the three population-based cohorts to that from NACC, in the youngest age stratum, cumulative incidence ranged across the three *APOE*-e4 doses from 1.46% to 5.60% in the population-based cohorts versus 7.94% to 23.06% in NACC, and in the oldest age stratum from 5.71% to 20.58% in the population-based cohorts versus 15.16% to 38.34% in NACC. Similarly, viewing the cumulative incidence for *APOE*-e4/e4 genotype across the three age strata, cumulative incidence ranged from 5.60% to 20.58% in the three population-based cohorts versus from 23.06% to 38.34% in NACC. The NACC findings were largely similar to those of the prospective analyses of Bonham et al. [24] in the same cohort, although Bonham et al. [24] focused on the *relative* risk of *APOE-*e4 across different age ranges, used different age categories (unrelated to the Generation Study), and did not incorporate several important variables in the models (i.e., family history, subjective memory concerns, and baseline cognitive performance). Moreover, the authors did not perform their analyses in a competing risk framework, which is vital to avoid overestimation of cumulative incidences in aging populations [54].

### Differences in cumulative incidence estimates across the sites

Variability related to ascertainment and assessment methods has been reported previously for MCI and dementia prevalence [55,56]. Such variability is not unique to MCI and dementia, but can occur in a variety of settings, and is a particular problem for common disorders like MCI in which a subtle gradation from the normal makes rates especially sensitive to thresholding (e.g., attention deficit hyperactivity disorder, major depression, osteoarthritis).

Overall, as might be expected, absolute risk is more vulnerable to methodological differences than relative risk, especially over shorter time intervals and for the MCI/dementia outcome rather than the dementia alone outcome. This is underscored by the generally similar relative hazards across the regression analyses. These regression findings also contribute to an understanding of the variation across the cohorts.

Among the three population-based cohort studies, there are known and unknown differences in race and ethnicity, education, and screening and assessment methods. Nonetheless, these three cohorts were generally similar—within expected sampling variation—in their estimates of cumulative incidence for most age and *APOE* strata. The difference between the population-based cohort studies and NACC, on the other hand, is striking. The NACC cohort is a volunteer cohort, and as such would not be expected to represent the general population (although it may be representative of potential trial participants, as discussed below). Individuals join this cohort for a variety of reasons, but concerns about family history and their own memory are likely to play a role. This probably contributes to the relatively high *APOE*-e4 allele frequency and reported family history of dementia in this cohort seen in Table 1, although some of the difference in family history likely represents measurement issues (see below). Since family history increases risk beyond the *APOE*-e4 effect in these and other data [57,58], the high frequency of positive family history likely contributed to some of the observed differences in incidence. Another potential source of difference is the very high level of educational attainment within the NACC cohort. While higher education is associated with lower risk of dementia overall, more educated individuals with memory concerns actually have higher risk of developing dementia than their counterparts with less education [59], and this may be particularly true for the highly educated individuals who form a substantial fraction of the NACC cohort. Another issue is the high proportion of women in the NACC cohort; differences in the reasons that men and women volunteer for this cohort may underlie the increased risk of MCI and dementia for men observed in the regression analyses. Last, the NACC samples serve a variety of needs across the different Alzheimer’s Disease Centers in the United States; there is often substantial dropout and variable effort to retain participants, and decisions by participants and center staff are not likely to be random with respect to cognitive and other variables. While the population-based cohort studies also have some dropout, systematic ongoing efforts to retain participants and continuous surveillance even for those who do not attend study visits guarantee low attrition.

Beyond these differences in ascertainment, demographics and other attributes, and follow-up, there are differences in assessment between NACC and the three population-based cohorts that should be noted. The population-based cohorts evaluate cognition with a screening procedure typically followed by more formal clinical evaluation of participants who screen positive. While direct clinical evaluation of all participants at each NACC site is a strength, there are procedural differences across sites, quality control is limited, and the reliability of NACC diagnosis is not well established. In addition, the high educational level of NACC participants is not well captured by available norms, and a subset of individuals may have declined substantially but nonetheless may be viewed as cognitively unimpaired. This is a particular concern because within any group of normal individuals, those who are already declining are more likely to continue to do so [60–62]. Thus, baseline cognitive symptoms and preexisting subthreshold decline, both likely to be more frequent in the highly educated NACC cohort, have a substantial impact on short-term onset of MCI and even dementia. This phenomenon may underlie the higher risk of cognitive decline noted earlier for more versus less educated individuals among those with subjective cognitive concerns [59].

Of course, it is likely that there is some insensitivity to MCI and even dementia in the population-based cohort studies as well as differential loss to follow-up, but on balance the volunteer nature of the NACC cohort, the limited quality control across the NACC sites, and the consistency of the population-based cohort findings tend to favor the lower cumulative incidence found in the population-based cohorts.

### Comparison to modeled estimates from the literature

One could argue that previously available modeled estimates for *APOE*-e4-associated absolute risk for dementia [17,19] are high (50%–67%), and thus favor the NACC estimates instead. Our estimates of lifetime risk for dementia for *APOE*-e4/e4 individuals from the Framingham Heart Study and the Rotterdam Study are in the 31%–40% range. While we did not estimate lifetime cumulative incidence for NACC given the short mean duration of follow-up, it would be expected to be considerably higher than the 5-y estimates—in the oldest *APOE*-e4/e4 homozygote individuals, 38.3% for MCI or dementia and 12.4% for dementia alone. However, there are some biases in the modeled estimates that overall are more likely to yield over- rather than underestimates of risk.

For the Cupples et al. estimates used in the REVEAL study [17], risk curves for incidence were derived from relatives and spouses in a family sample ascertained from a clinical population [18]; these incidence rates could be expected to be higher than those in the general population. In addition, the relative risks by sex, age, and genotype were applied from a large meta-analysis done primarily in clinically ascertained, younger-onset families [14], again yielding higher estimates [14,63]. In addition, the competing risk of death was not addressed in the cumulative incidence estimates, which also would tend to bias estimates upward. Moreover, the Cupples model does not account for the correlation among observations in the family sample used for incidence, which again might lead to bias [64].

For the Genin et al. estimates used by 23andMe [19], relative risks from a European GWAS [21] were applied to incidence estimates from the Rochester [22] and PAQUID [23] cohorts. The relative risk estimates come from cases and controls, with younger cases (with a greater *APOE*-e4 effect) overrepresented. In addition, these models assumed that the controls in GWAS samples were representative of the overall population. This likely does not hold with a very common disease like dementia (which occurs in over 10% of those over 65 y and 35% or more of those over 85 y [1]) because at higher ages those without dementia are fundamentally a selected sample. This also would tend to bias the estimates upward.

### Insights from the regression models

Overall, the substantial effects of age and *APOE*-e4 dose were consistent across the univariable and basic and more complex multivariable models, persisting even when other demographic factors as well as cognitive variables and family history were taken into account. Education also exhibited a dose response, but behaved less consistently, as much illustrating as illuminating the profound differences in education across these four cohorts.

The effect of sex is even less consistent, perhaps reflecting ascertainment and cultural differences across disparate cohorts; findings in the literature are also inconsistent [23,65,66]. Some studies suggest also that *APOE*-e4 behaves differently by sex, with a greater effect in women [67,68]. If we had had sufficient sample size, we would also have stratified our risk estimates on sex or considered including an interaction term in our regression models. However, in the population-based cohorts for the MCI/dementia outcome, there was strong attenuation of the effect estimates of sex when adjusting for educational attainment, suggesting that lack of educational attainment in women of older birth cohorts may partly explain the difference. However, for dementia only in the same cohorts, a nominally statistically significant higher risk in women persisted even after adjustment for demographics and other risk indicators. Conversely, in NACC, there was a higher risk in men, which we believe is likely related to ascertainment differences by sex in this convenience sample, as noted above. Overall, potential sex differences deserve particular focus in future studies given the complex relationships among sex, education, vascular risk factors, birth cohort, longevity, and genetics.

Also of potential relevance, both to potential participants wishing to understand their absolute risk and to investigators designing clinical trials, both cognitive performance and subjective memory concerns were associated with an increased hazard of MCI or dementia. All in all, these associations suggest that relatively simple individual characteristics might be used to further refine individual risk stratification beyond age and *APOE* genotype.

### Implications for study design and genetic counseling

For the purposes of the Generation Study and other prevention trials, absolute cumulative incidence, both during the 5-y duration of the trial and over the remaining lifetime, is critical, but the differences across these cohort studies make it difficult to offer precise estimates, even with meta-analyses. In an ideal world, estimates would be tailored to the population entering the trial or, better still, the specific individuals, and would take into account not only explicit inclusion criteria but also any other measureable or predictable characteristics that might predict willingness to volunteer. A review of the first registrants on the GeneMatch registry, which serves as the primary US recruiting site for the Generation Study *APOE*-e4/e4 trial, shows that registrants differ from the general population beyond the explicit entry criteria. The population of 13,704 registrants enrolled thus far is relatively young (mean age 62.7 y, standard deviation 5.2) and women are overrepresented (80%). Among the 4,978 registrants who were asked about race/ethnicity, 92% are white. The frequency of the *APOE*-e4/e4 genotype among registrants is higher than in the general population, at 4.47%, and the *APOE*-e4 allele frequency is 20.4%; among the 3,456 registrants asked about whether they had a family history of dementia or Alzheimer disease, 70.1% said yes. While education was not measured, the high percent of females and individuals with a significant family history (and the high *APOE-*e4 frequency) suggests a population that may be more like NACC. However, data on education, cognitive performance, and subjective memory concerns are not available. Moreover, over time, if there are broader recruiting efforts in order to reach the target sample size, volunteers could gradually become more reflective of the general population, and lower risks might be expected.

In the genetic counseling setting, any risk information would need to give a broad range of estimates to reflect uncertainty within cohorts and variation across cohorts. Because risk for disease is ongoing, and the lifetime risks were more stable than the 5-y risks in our analyses, we thought the lifetime risks were more informative for genetic disclosure. However, such risks may be less salient to some of those considering enrollment in trials at younger ages. The Generation Study elected to disclose the following “lifetime” risks of MCI or dementia to its potential participants: 30%–55% for individuals with *APOE*-e4/e4; 20%–25% for individuals with *APOE*-e3/e4 and -e2/e4 (with a note that risk might be lower for those with *APOE-*e2/e4); and 10%–15% for individuals with *APOE*-e3/e3, -e3/e2, and -e2/e2 (with a note that risk might be lower for those with *APOE*-e2/e3 and -e2/e2). These values are consistent with our findings, but use round numbers for intelligibility, and broader ranges to reflect statistical and other sources of uncertainty. The regression models are insufficiently precise for “personalized medicine” incidence estimates based on sex, education, or other factors, but they do allow for qualitative adjustments to overall stratified risk estimates. Relative risks by *APOE* genotype or *APOE*-e4 dose have limited relevance in the setting of the prevention trial, but may provide context. If these are provided, risk should be compared to the general population (based on a weighted average across the three possible *APOE*-e4 doses rather than the typical “no *APOE*-e4” base category used in regression models), which would more fairly allow a participant to put his or her own risk in the context of friends and acquaintances of unknown genotype. On the basis of our regression findings (S1 Appendix Table E), for *APOE-*e4/e4 homozygotes, the adjusted relative risk for MCI/dementia is 2.7 for NACC, 3.4 for the Framingham Heart Study, and 2.4 for the Rotterdam Study, so disclosing a relative risk of about 3-fold compared to the general population would make sense. Use of pictographs as a visual aid for risk communication could be useful, given their ability to visually represent both absolute and relative risk information simultaneously [69]. In addition, there is a robust literature on genetic risk communication that can inform best practices in cases where *APOE* information is disclosed to asymptomatic individuals [70].

### Limitations

One major limitation of this study is that *APOE*-e4/e4 samples are small despite the large size of the initial cohorts, particularly for SALSA. This limits the stability of stratified cumulative incidence estimates (only partially addressed by the meta-analyses) as well as regression coefficients for *APOE*-e4 dose. This issue is further complicated by missing data (likely not missing at random) and likely differential dropout. Second, while the four cohorts are heterogeneous in sex distribution and education, there is little ethnic and racial diversity, so the findings are less relevant to participants of non-European background. Third, variations in the definitions of the exposure and outcome variables may hamper comparisons among cohorts. As noted above, each cohort uses different criteria to define unimpaired at baseline, and to screen, assess, and diagnose new onset cases. Different psychometric tests are applied, and even the same test performs differently across different groups; education- and/or age-adjusted norms can compensate for this, but may introduce other problems in interpretation. Other variation may come from differences in definitions (e.g., family history is based on a single question about parents only in the Rotterdam Study versus a detailed set of questions about each parent and sibling in NACC) or in how information is acquired (being positive for memory concerns is based on a yes answer to any one of three questionnaire items in the Rotterdam Study versus an overall clinical impression about the participant’s attitude in NACC). Moreover, some variables, notably level of education, may be defined similarly but have different meanings within different cultural contexts. Nevertheless, as we have shown, relative risk estimates are consistent despite this variation. Fourth, regression models for MCI or dementia are limited because of confounding and omitted predictors, and are complicated by multicollinearity of exposure and outcome variables that represents confounding, effect modification, and true signal.

### Conclusion

Prospective cohort studies can be used to inform study design, power, and informed consent in clinical trials among cognitively unimpaired individuals. While trial designers and participants may be most interested in absolute risk over relatively short intervals, absolute risk is less robustly estimated than relative risk, and short-term risk less robustly estimated (and more sensitive to the definition and operationalization of cognitively unimpaired at baseline) than long-term risk. Estimation that serves informed consent and optimal trial design will require matching the cohort used to estimate risk as closely as possible to trial participants.

Overall, the estimates for *APOE*-associated risk of MCI or dementia were lower in our study than previously reported, and there is reason to believe that the risk estimates obtained in the population-based cohorts more accurately reflect the general population than those obtained in NACC. However, these lower risks may less accurately match the likely trial population. In general, such estimates are also sensitive to variation in sampling, assessment, and modeling. Rigorous attention to sampling, assessment, and statistical methods is critical to developing the best possible answers for clinical trial design.

## Supporting information

S1 Checklist STROBE statement checklist with paragraph numbers per section. (DOC)

S1 Appendix Supplementary tables of results for subdistribution hazard regression analyses. (DOCX)

S1 Text Funding information. (DOCX)

S2 Text Data availability statement. (DOCX)

## Abbreviations

3MS: Modified Mini-Mental State Examination
GWAS: genome-wide association study
HR: hazard ratio
IRB: institutional review board
MCI: mild cognitive impairment
MMSE: Mini-Mental State Examination
NACC: National Alzheimer’s Coordinating Center
PAQUID: Personnes Agées QUID
REVEAL: Risk Evaluation and Education for Alzheimer’s Disease
SALSA: Sacramento Area Latino Study on Aging
