# Supplementary material for: APOE-related risk of mild cognitive impairment and dementia for prevention trials: An analysis of four cohorts
Source: PLoS Med. 2017 Mar 21;14(3):e1002254. doi: 10.1371/journal.pmed.1002254 (PMC5360223; doi:10.1371/journal.pmed.1002254)
Supplement: S1 Appendix — (DOCX) [file pmed.1002254.s002.docx]

Table A:  Subdistribution hazard regression for mild cognitive impairment /dementia: univariable analyses.

| **Characteristic** | **Cohort** | | | |
| --- | --- | --- | --- | --- |
|  | **NACC** | **RS** | **FHS**^#^ | **SALSA** |
|  | **Hazard ratio**  **(95% CI)** | **Hazard ratio**  **(95% CI)** | **Hazard ratio**  **(95% CI)** | **Hazard ratio**  **(95% CI)** |
| **Age (years) at baseline visit** | 1.08  (1.06, 1.10) | 1.09  (1.08, 1.10) | 1.16  (1.14,1.18) | 1.10  (1.06, 1.15) |
| **Male sex** | 1.36  (1.16,1.60) | 0.83  (0.74, 0.93) | 0.89  (0.76, 1.04) | 0.90  (0.61,1.13) |
| ***APOE*-e4 dose (reference: 0 e4)** |  |  |  |  |
| 1 e4 | 1.51  (1.27, 1.78) | 1.64  (1.46, 1.84) | 1.57  (1.32, 1.88) | 2.23  (1.45, 3.43) |
| 2 e4 | 2.79  (1.98, 3.92) | 2.63  (2.02, 3.42) | 3.30  (2.08, 5.22) | 3.57  (1.19, 10.70) |
| **Education (reference: High school)** |  |  |  |  |
| Less than high school | 1.86  (1.24, 2.80) | 1.41  (1.21, 1.64) | 1.80  (1.46, 2.22) | 1.54  (0.86, 2.75) |
| Some college | 0.83  (0.62, 1.11) | 0.81  (0.71, 0.93) | 1.05  (0.85, 1.29) | 0.99  (0.41, 2.39) |
| College graduation | 0.85  (0.67, 1.06) | 0.56  (0.46, 0.69) | 0.76  (0.60, 0.96) | 1.37  (0.62, 3.02) |
| **Standardized cognitive screening test score, per SD**^‡^ | 0.62  (0.57, 0.66) | 1.00  (0.95, 1.06) | 0.80  (0.76, 0.85) | 0.58  (0.52, 0.65) |
| **Subjective memory concerns** | 2.62  (2.22, 3.08) | 1.71  (1.53, 1.91) | N/A | N/A |
| **Family History of dementia** | 1.16  (0.98, 1.37) | 1.27  (1.11, 1.44) | N/A | N/A |
| **Vascular risk score, (reference: Moderate)** ^$^ |  |  |  |  |
| Low | 0.74  (0.60, 0.90) | 0.88  (0.65, 1.20) | 1.36  (1.13, 1.63) | 0.53  (0.17, 1.68) |
| High | 1.05  (0.98, 1.37) | 0.97  (0.85, 1.12) | 0.83  (0.63, 1.11) | 1.44  (0.98, 2.12) |

^#^For FHS, first visit after age 60 with available MMSE was used as the baseline.

^‡^MMSE for NACC, the Rotterdam Study, and the Framingham Heart Study; in SALSA, the 3MS was used; all tests standardized *within* the sample in question.

^$^Vascular risk score was calculated based on a count of standard risk factors, see text for details.

3MS, Modified Mini-Mental State Examination; MMSE, Mini-Mental State Examination; N/A, not available; NACC, National Alzheimer’s Coordinating Center; RS, Rotterdam Study; FHS, Framingham Heart Study; SALSA, Sacramento Area Latino Study on Aging.

Table B:  Subdistribution hazard regression for dementia:  univariable analyses.

| **Characteristic** | **Cohort** | | | |
| --- | --- | --- | --- | --- |
|  | **NACC** | **RS** | **FHS**^#^ | **SALSA** |
|  | **Hazard ratio**  **(95% CI)** | **Hazard ratio**  **(95% CI)** | **Hazard ratio**  **(95% CI)** | **Hazard ratio**  **(95% CI)** |
| **Age (years) at baseline visit** | 1.22  (1.13, 1.32) | 1.12  (1.10, 1.14) | 1.16  (1.14, 1.19) | 1.19  (1.11, 1.28) |
| **Male sex** | 1.26  (0.73,2.17) | 0.68  (0.59, 0.79) | 0.78  (0.65, 0.93) | 0.84  (0.47,1.49) |
| ***APOE*-e4 dose (reference: 0 e4)** |  |  |  |  |
| 1 e4 | 1.98  (1.11, 3.53) | 2.07  (1.79, 2.40) | 1.66  (1.36, 2.02) | 3.42  (1.87, 6.24) |
| 2 e4 | 8.37  (3.89, 18.02) | 4.88  (3.61, 6.59) | 3.19  (1.86, 5.45) | 5.82  (1.53, 22.13) |
| **Education (reference: High school)** |  |  |  |  |
| Less than high school | 3.35  (1.09, 10.31) | 1.34  (1.10, 1.62) | 1.70  (1.35, 2.15) | 1.57  (0.67, 3.69) |
| Some college | 0.89  (0.34, 2.37) | 0.82  (0.69, 0.97) | 0.95  (0.75, 1.21) | 0.82  (0.20, 3.25) |
| College graduation | 0.86  (0.40, 1.85) | 0.64  (0.49, 0.84) | 0.71  (0.54, 0.93) | 0.80  (0.20, 3.20) |
| **Standardized cognitive screening test score, per SD**^‡^ | 0.69  (0.59, 0.80) | 0.90  (0.83, 0.96) | 0.82  (0.77, 0.88) | 0.50  (0.43, 0.59) |
| **Subjective memory complaints** | 1.68  (0.96, 2.95) | 1.47  (1.28, 1.69) | N/A | N/A |
| **Family history of dementia** | 1.57  (0.87, 2.85) | 1.44  (1.22, 1.69) | N/A | N/A |
| **Vascular risk score (reference level: Moderate)** ^$^ |  |  |  |  |
| Low | 0.80  (0.42, 1.50) | 0.83  (0.56, 1.24) | 1.33  (1.08, 1.64) | 0.52  (0.17, 1.63) |
| High | 0.60  (0.24, 1.53) | 0.78  (0.65, 0.95) | 0.82  (0.59, 1.13) | 1.40  (0.95, 2.06) |

^#^For FHS, first visit after age 60 with available MMSE was used as the baseline.

^‡^MMSE for NACC, the Rotterdam Study, and the Framingham Heart Study; in SALSA, the 3MS was used; all tests standardized *within* the sample in question.

^$^Vascular risk score was calculated based on a count of standard risk factors, see text for details.

3MS, Modified Mini-Mental State Examination; MMSE, Mini-Mental State Examination; N/A, not available; NACC, National Alzheimer’s Coordinating Center; RS, Rotterdam Study; FHS, Framingham Heart Study; SALSA, Sacramento Area Latino Study on Aging.

Table C: Subdistribution hazard regression analyses for mild cognitive impairment/dementia; *APOE* and demographics.

| **Characteristic** | **Cohort** | | | |
| --- | --- | --- | --- | --- |
|  | **NACC** | **RS** | **FHS**^#^ | **SALSA** |
|  | **Hazard ratio**  **(95% CI)** | **Hazard ratio**  **(95% CI)** | **Hazard ratio**  **(95% CI)** | **Hazard ratio**  **(95% CI)** |
| **Age (years) at baseline visit** | 1.08  (1.06, 1.10) | 1.09  (1.07, 1.10) | 1.15  (1.13, 1.17) | 1.10  (1.05, 1.14) |
| **Male sex** | 1.40  (1.19,1.65) | 0.92  (0.82, 1.04) | 0.96  (0.81, 1.13) | 0.85  (0.57,1.26) |
| ***APOE*-e4 dose (reference: 0 e4)** |  |  |  |  |
| 1 e4 | 1.58  (1.33, 1.87) | 1.65  (1.47, 1.85) | 1.72  (1.43, 2.07) | 2.26  (1.47, 3.48) |
| 2 e4 | 3.35  (2.39, 4.69) | 2.89  (2.21, 3.77) | 4.10  (2.42, 6.97) | 4.13  (1.38, 12.40) |
| **Education (reference: High school)** |  |  |  |  |
| Less than high school | 1.85  (1.23, 2.79) | 1.20  (1.03, 1.41) | 1.44  (1.16, 1.78) | 1.39  (0.77, 2.49) |
| Some college | 0.86  (0.64, 1.15) | 0.83  (0.73, 0.95) | 1.07  (0.87, 1.33) | 0.96  (0.40, 2.32) |
| College graduation | 0.83  (0.66, 1.04) | 0.61  (0.49, 0.76) | 0.83  (0.65, 1.06) | 1.41  (0.63, 3.15) |

^#^For FHS, first visit after age 60 with available MMSE was used as the baseline.

NACC, National Alzheimer’s Coordinating Center; RS, Rotterdam Study; FHS, Framingham Heart Study; SALSA, Sacramento Area Latino Study on Aging.

Table D: Subdistribution hazard regression analyses for dementia: *APOE* and demographics.

| **Characteristic** | **Cohort** | | | |
| --- | --- | --- | --- | --- |
|  | **NACC** | **RS** | **FHS**^#^ | **SALSA** |
|  | **Hazard ratio**  **(95% CI)** | **Hazard ratio**  **(95% CI)** | **Hazard ratio**  **(95% CI)** | **Hazard ratio**  **(95% CI)** |
| **Age (years) at baseline visit** | 1.24  (1.15, 1.35) | 1.12  (1.10, 1.14) | 1.15  (1.12, 1.18) | 1.19  (1.11, 1.27) |
| **Male sex** | 1.33  (0.78,2.27) | 0.72  (0.62, 0.84) | 0.84  (0.69, 1.02) | 0.80  (0.45,1.44) |
| ***APOE*-e4 dose (reference: 0 e4)** |  |  |  |  |
| 1 e4 | 2.21  (1.23, 3.96) | 2.11  (1.81, 2.45) | 1.86  (1.52, 2.29) | 3.44  (1.89, 6.27) |
| 2 e4 | 11.91  (5.45, 26.00) | 5.60  (4.10, 7.66) | 3.91  (2.13, 7.16) | 7.23  (1.85, 28.29) |
| **Education (reference: High school)** |  |  |  |  |
| Less than high school | 3.87  (1.22, 11.89) | 1.11  (0.91, 1.36) | 1.38  (1.08, 1.75) | 1.31  (0.57, 3.03) |
| Some college | 1.00  (0.38, 2.66) | 0.88  (0.74, 1.05) | 0.95  (0.74, 1.21) | 0.72  (0.18, 2.90) |
| College graduation | 0.89  (0.41, 1.91) | 0.78  (0.59, 1.03) | 0.77  (0.58, 1.02) | 0.83  (0.21, 3.23) |

^#^For FHS, first visit after age 60 with available MMSE was used as the baseline.

NACC, National Alzheimer’s Coordinating Center; RS, Rotterdam Study; FHS, Framingham Heart Study; SALSA, Sacramento Area Latino Study on Aging.

Table E: Subdistribution hazard regression analyses for mild cognitive impairment /dementia: *APOE*, demographics, cognition, and family history.

| **Characteristic** | **Cohort** | | | |
| --- | --- | --- | --- | --- |
|  | **NACC** | **RS** | **FHS^#^** | **SALSA** |
|  | **Hazard ratio**  **(95% CI)** | **Hazard ratio**  **(95% CI)** | **Hazard ratio**  **(95% CI)** | **Hazard ratio**  **(95% CI)** |
| **Age (years) at baseline visit** | 1.08  (1.05, 1.10) | 1.08  (1.07, 1.09) | 1.15  (1.12, 1.17) | 1.07  (1.03, 1.12) |
| **Male sex** | 1.14  (0.96,1.36) | 0.92  (0.81, 1.03) | 0.93  (0.79, 1.10) | 0.84  (0.56,1.25) |
| ***APOE*-e4 dose (reference: 0 e4)** |  |  |  |  |
| 1 e4 | 1.49  (1.25, 1.79) | 1.63  (1.44, 1.84) | 1.75  (1.45, 2.10) | 2.15  (1.39, 3.33) |
| 2 e4 | 2.37  (1.59, 3.53) | 2.78  (2.10, 3.69) | 4.01  (2.31, 6.96) | 1.65  (0.27, 9.93) |
| **Education (reference: High school)** |  |  |  |  |
| Less than high school | 1.41  (0.91, 2.19) | 1.24  (1.06, 1.46) | 1.33  (1.06, 1.65) | 0.80  (0.43, 1.49) |
| Some college | 0.90  (0.66, 1.22) | 0.83  (0.72, 0.95) | 1.10  (0.89, 1.36) | 1.01  (0.42, 2.43) |
| College graduation | 0.92  (0.73, 1.16) | 0.62  (0.50, 0.77) | 0.87  (0.69, 1.11) | 1.61  (0.72, 3.62) |
| **Standardized cognitive screening test score, per SD** ^‡^ | 0.63  (0.58, 0.69) | 1.08  (1.02, 1.15) | 0.87  (0.82, 0.93) | 0.59  (0.52, 0.67) |
| **Subjective memory concerns** | 2.23  (1.87, 2.66) | 1.56  (1.39, 1.74) | N/A | N/A |
| **Family history of dementia**^§^ | 1.27  (1.06, 1.52) | 1.16  (1.01, 1.32) | N/A | N/A |

^#^For FHS, first visit after age 60 with available MMSE was used as the baseline.

^‡^ MMSE for NACC, the Rotterdam Study, and the Framingham Heart Study; in SALSA, the 3MS was used; all tests standardized *within* the sample in question.

^§^Family history of dementia was not available in the SALSA and Framingham Heart Study cohorts.

3MS, Modified Mini-Mental State Examination; MMSE, Mini-Mental State Examination; N/A, not available; NACC, National Alzheimer’s Coordinating Center; RS, Rotterdam Study; FHS, Framingham Heart Study; SALSA, Sacramento Area Latino Study on Aging.

Table F: Subdistribution hazard regression analyses for dementia*: APOE*, demographics, cognition, and family history.

| **Characteristic** | **Cohort** | | | |
| --- | --- | --- | --- | --- |
|  | **NACC** | **RS** | **FHS**^#^ | **SALSA** |
|  | **Hazard ratio**  **(95% CI)** | **Hazard ratio**  **(95% CI)** | **Hazard ratio**  **(95% CI)** | **Hazard ratio**  **(95% CI)** |
| **Age (years) at baseline visit** | 1.24  (1.14, 1.34) | 1.11  (1.09, 1.13) | 1.14  (1.12, 1.17) | 1.14  (1.07, 1.22) |
| **Male sex** | 1.18  (0.65, 2.15) | 0.72  (0.61, 0.85) | 0.81  (0.67, 0.99) | 0.79  (0.44, 1.42) |
| ***APOE*-e4 dose (reference: 0 e4)** |  |  |  |  |
| 1 e4 | 2.39  (1.30, 4.39) | 2.09  (1.79, 2.44) | 1.90  (1.54, 2.33) | 3.46  (1.92, 6.23) |
| 2 e4 | 8.39  (3.28, 21.44) | 5.31  (3.85, 7.34) | 3.90  (2.11, 7.22) | 1.97  (0.19, 20.93) |
| **Education (reference: High school)** |  |  |  |  |
| Less than high school | 3.76  (1.17, 12.06) | 1.08  (0.88, 1.33) | 1.28  (1.00, 1.63) | 0.63  (0.26, 1.55) |
| Some college | 1.14  (0.43, 3.06) | 0.87  (0.73, 1.04) | 0.97  (0.76, 1.24) | 0.69  (0.17, 2.91) |
| College graduation | 0.89  (0.40, 1.98) | 0.76  (0.57, 1.02) | 0.81  (0.61, 1.07) | 0.97  (0.25, 3.78) |
| **Standardized cognitive screening test score, per SD** ^‡^ | 0.73  (0.59, 0.90) | 0.96  (0.89, 1.04) | 0.88  (0.83, 0.94) | 0.53  (0.43, 0.66) |
| **Subjective memory concerns** | 1.06  (0.56, 1.99) | 1.25  (1.08, 1.44) | N/A | N/A |
| **Family history of dementia**^§^ | 1.83  (0.96, 3.50) | 1.29  (1.09, 1.52) | N/A | N/A |

^#^For FHS, first visit after age 60 with available MMSE was used as the baseline.

^‡^ MMSE for NACC, the Rotterdam Study, and the Framingham Heart Study; in SALSA, the 3MS was used; all tests standardized *within* the sample in question.

^§^Family history of dementia was not available in the SALSA and Framingham Heart Study cohorts.

3MS, Modified Mini-Mental State Examination; MMSE, Mini-Mental State Examination; N/A, not available; NACC, National Alzheimer’s Coordinating Center; RS, Rotterdam Study; FHS, Framingham Heart Study; SALSA, Sacramento Area Latino Study on Aging.
