# Supplementary material for: APOE-related risk of mild cognitive impairment and dementia for prevention trials: An analysis of four cohorts
Source: PLoS Med. 2017 Mar 21;14(3):e1002254. doi: 10.1371/journal.pmed.1002254 (PMC5360223; doi:10.1371/journal.pmed.1002254)
Supplement: S1 Text — (DOCX) [file pmed.1002254.s003.docx]

**Funding Information**

This study was primarily funded by the National Institute on Aging (1UF1AG046150; Reiman and Tariot, PIs): EMR, PNT, JBL, DB, RAB, JQ, MAI, FW, SS, AB, MH, JMN, JK, JSR, BMW. Additional funding was received from the National Institute on Aging: P50AG005134 (DB, RAB), R21AG053695 (JQ, RAB), R01 AG12975 (MH, JMN), AG054076 (SS, AB), R01 AG008122 (SS, AB), R01 AG049607 (SS, AB), R01 AG033193 (SS, AB), U01 AG049505 (SS, AB); the National Institute for Neurological Diseases and Stroke: R01NS094610 (RAB, JQ), NS017950 (SS, AB), UH2 NS100605 (SS, AB); the National Heart Lung and Blood Institute: contract #HHSN268201500001I (SS, AB); and the European Research Council (ERC) under the European Union’s Horizon 2020 research and innovation programme project: ORACLE, grant agreement No: 678543 (MAI); and the Harvard NeuroDiscovery Center: no grant number (RAB, JQ). The NACC database is funded by NIA/NIH Grant U01 AG016976. NACC data are contributed by the NIA-funded ADCs: P30 AG019610 (PI Eric Reiman, MD), P30 AG013846 (PI Neil Kowall, MD), P50 AG008702 (PI Scott Small, MD), P50 AG025688 (PI Allan Levey, MD, PhD), P50 AG047266 (PI Todd Golde, MD, PhD), P30 AG010133 (PI Andrew Saykin, PsyD), P50 AG005146 (PI Marilyn Albert, PhD), P50 AG005134 (PI Bradley Hyman, MD, PhD), P50 AG016574 (PI Ronald Petersen, MD, PhD), P50 AG005138 (PI Mary Sano, PhD), P30 AG008051 (PI Steven Ferris, PhD), P30 AG013854 (PI M. Marsel Mesulam, MD), P30 AG008017 (PI Jeffrey Kaye, MD), P30 AG010161 (PI David Bennett, MD), P50 AG047366 (PI Victor Henderson, MD, MS), P30 AG010129 (PI Charles DeCarli, MD), P50 AG016573 (PI Frank LaFerla, PhD), P50 AG016570 (PI Marie-Francoise Chesselet, MD, PhD), P50 AG005131 (PI Douglas Galasko, MD), P50 AG023501 (PI Bruce Miller, MD), P30 AG035982 (PI Russell Swerdlow, MD) , P30 AG028383 (PI Linda Van Eldik, PhD), P30 AG010124 (PI John Trojanowski, MD, PhD), P50 AG005133 (PI Oscar Lopez, MD), P50 AG005142 (PI Helena Chui, MD), P30 AG012300 (PI Roger Rosenberg, MD), P50 AG005136 (PI Thomas Montine, MD, PhD), P50 AG033514 (PI Sanjay Asthana, MD, FRCP), P50 AG005681 (PI John Morris, MD), and P50 AG047270 (PI Stephen Strittmatter, MD, PhD). The authors also wish to note that Alzheimer Prevention Initiative Generation Study Clinical Trial is also funded by Novartis and Amgen (JBL, EMT, EMR). The funders had no role in study design, data collection and analysis, decision to publish, or preparation of the manuscript.
