# Supplementary material for: APOE-related risk of mild cognitive impairment and dementia for prevention trials: An analysis of four cohorts
Source: PLoS Med. 2017 Mar 21;14(3):e1002254. doi: 10.1371/journal.pmed.1002254 (PMC5360223; doi:10.1371/journal.pmed.1002254)
Supplement: S2 Text — (DOCX) [file pmed.1002254.s004.docx]

**DATA AVAILABILITY STATEMENT**

Qian et al, *APOE* and risk of MCI or dementia for prevention trials

Individual level data for the Framingham Heart Study is available through dbGaP (https://www.ncbi.nlm.nih.gov/projects/gap/cgi-bin/study.cgi?study_id=phs000007.v28.p10) or through the General/Limited Access Datasets of NHLBI or through a Data and Materials Distribution Agreement (DMDA) with the Framingham investigators (http://www.framinghamheartstudy.org/researchers/application-review.php).

Rotterdam Study data can be made available to interested researchers upon request. Requests can be directed to data manager Frank J.A. van Rooij (f.vanrooij@erasmusmc.nl). We are unable to place data in a public repository due to legal and ethical restraints. Sharing of individual participant data was not included in the informed consent of the study, and there is potential risk of revealing participants’ identities as it is not possible to completely anonymize the data. This is of particular concern given the sensitive personal nature of much of the data collected as part of the Rotterdam Study.

Virtually all of the SALSA data is archived at the University of Michigan under the National Archive for Computerized Data on Aging (NACDA) program( http://www.icpsr.umich.edu/icpsrweb/NACDA/studies/29323). SALSA investigators are currently updating the NACDA website with new variables and recent assessments.

To obtain the NACC data, contact NACC via their website ([www.alz.washington.edu](http://www.alz.washington.edu/)) or email ([consnacc@uw.edu](https://mail.nmr.mgh.harvard.edu/webmail/images/blank.png)), and indicate that you wish the file 'blacker1004016.csv' used in the present publication.

Coding scripts for the analysis are available from the first author upon request ([qian@schoolph.umass.edu](mailto:qian@schoolph.umass.edu)).
